# Combination of mesenchymal stem cell injection with icariin for the treatment of diabetes-associated erectile dysfunction

**DOI:** 10.1371/journal.pone.0174145

**Published:** 2017-03-28

**Authors:** Xiyou Wang, Chuanhai Liu, Yong Xu, Ping Chen, Yue Shen, Yansheng Xu, Yubo Zhao, Weihao Chen, Xinyu Zhang, Yun Ouyang, Yi Wang, Changliang Xie, Maojun Zhou, Cuilong Liu

**Affiliations:** 1 Department of Urology, PLA Navy General Hospital, Hai dian District, Beijing, People's Republic of China; 2 Department of Urology, The Second Artillery General Hospital of Chinese People’s Liberation Army, Xicheng District, Beijing, People's Republic of China; 3 Department of Urology, PLA General Hospital, Hai dian District, Beijing, People's Republic of China; Nanjing Medical University, CHINA

## Abstract

The present study was aimed to examine whether icariin, a traditional Chinese medicine, could improve therapeutic effects of adipose derived mesenchymal stem cells (ADSCs) for diabetes-associated erectile dysfunction (DMED). DMED were induced in rats by intraperitoneal injection of streptozotocin and confirmed by erectile function measurement. Then, rats of diabetic ED were randomly divided to receive the treatment of saline, ADSCs, icariin or ADSCs combined with icariin respectively. Compared with the treatment by ADSCs or icariin alone, intracavernosum injection of ADSCs combined with the following daily gastric gavage of icariin significantly augmented the value of ICP and ICP/MAP (p<0.01). Meanwhile, the survival of transplanted ADSCs was much improved due to the application of icariin. Similarly, immunofluorescent staining analysis demonstrated that the improved erectile tissue structure by combination of ADSCs and icariin was significantly associated with the increased expression of endothelial markers (vWF) (p<0.01) and smooth muscle markers (α-SMA) (p<0.01). Furthermore, the structure changes in corpus cavernosum were further confirmed by the Masson’s trichrome staining. To explore the possible mechanism underlying icariin-enhanced therapeutic efficacy of MSCs, we employed an *in vitro* testing system by introducing H_2_O_2_ to imitate oxidative stress condition considering the oxidative environment faced by engrafted ADSCs and anti-oxidative capacity of icariin_._ *In vitro*, we found that the addition of icariin considerably reduced the apoptosis of ADSCs, and attenuated the intracellular reactive oxygen species (ROS), the superoxidase dismutase (SOD) activity and the lactate dehydrogenase (LDH). Subsequently, we examined the expression of apoptosis-related proteins and explored the potential signaling pathway through which icariin promoted the survival of ADSCs against oxidative stress. It was demonstrated that icariin significantly inhibited the upregulation of apoptosis-related proteins under oxidative condition, including Bax and cleaved caspase-3, while promoted the expression of anti-apoptotic factor BCL2. These effects were accompanied with the activation of signal molecules, PI3K/Akt and STAT3. The further signal protein inhibition assays exhibited that the suppression of STAT3 abrogated the icariin-mediated anti-apoptotic effects observed above, while did not influence the expression of PI3K/Akt. However, PI3K inhibition could abrogate icariin–mediated STAT3 activation and achieved a similar effect as STAT3 inhibition. Our results suggested that icariin was an effective adjuvant for enhancing ADSC-based therapy of DMEM, which may be ascribed to their protection of ADSCs against oxidative stress via the regulation of PI3K/Akt-STAT3 signal pathway.

## Introduction

Erectile dysfunction (ED), otherwise known as impotency in men, has an impact on over 30 million men every year, threatening human health and life. In modern life, various risk factors contribute to the incidence of ED among which diabetes mellitus (DM) is showed to be highly associated. Generally, diabetic male patients were addressed to possess a higher ratio of suffering ED than the non-diabetic men [1, 2]. Complicated causes contribute to the incidence of ED. In brief, functional impairments in blood vessel, muscle, and nerve [3] possess the main responsibility. Otherwise, DM was able to produce oxidative stress damage in cavernoursal tissues, usually resulting in the loss of the physiological properties of endothelium and shifting to a vasoconstrictor, pro-thrombotic and pro-inflammatory state [4, 5], which is considered to be crucial in the early development of diabetic-associated erectile dysfunction (DMED). Moreover, cavernoursal smooth muscles are also found to lose their function in DMED, along with the reduction of low density lipoprotein (LDL) and the augment of reactive oxygen species (ROS) [6].

In the past years, abundant studies were performed in the development of oral medications for DMED, such as the commonly prescribed phosphodiesterase type 5 (PDE5) inhibitors [7, 8]. However, the PDE5 inhibitor was not effective among patients with severe erectile dysfunction [9]. Additionally, common side effects of these drugs, including headache, flushing, dyspepsia, nasal congestion, abnormal vision and diarrhea, now and then attacked the patients who accepted the drug-based treatments [3]. In addition to drugs, other optional strategies, such as vacuum constriction devices and penile prosthesis implantation, are available too, but the efficiency was usually limited due to the lack of restoration of cells and tissues in both function and structure [3, 10]. Therefore, the development of a novel therapy method which is able to overcome aforementioned drawbacks in the DMED therapy is considerably required.

Currently, stem cell-based therapies attract growing attentions for their capability of facilitating functional recovery and tissue structural repair for DMED patients. Among a variety of stem cells, mesenchymal stem cells (MSCs) are evidenced to be one of the most promising candidates for DMED due to their abundant autologous availability [11]. More importantly, allogeneic MSCs can also achieve successful transplantation [12] as the result of a low immunogenicity of the cells [13], which largely extends the usage of MSCs. As for ED therapy, MSCs implantation has been proved to significantly improve the recovery of erectile function in animal studies [14] by inhibiting apoptosis [15]. Focusing on the diabetic ED, MSCs based treatment also received encouraging therapeutic effects [16]. And the restored erectile function is mainly attributed for the increasing contents of endothelium and smooth muscle in corpus cavernosum [17, 18]. Thus, having established the feasibility of MSCs for DMED treatment, recently, various strategies have emerged for the purpose of improving the therapeutic effects of MSCs for DMED [19–21]. Previously, we reported that hypoxic preconditioning (HP) was able to promote MSCs based treatment of ED via strengthening the therapeutic ability of individual cells, such as the reinforced paracrine function [22].

Icariin, a major flavonoid isolated from the traditional oriental herbal medicine Epimedium koreanum Nakai [23], received our particular attention in recent years for its broad bioactivities and applications, such as its anti-oxidative capacity, cardio-protective effects, and immunological function regulation [24–27]. Icariin had been applicated in the treatment of ED though the mechanisms were still ambiguous, suggesting the feasibility of applying icariin for the MSCs-based ED therapy [28].Inspired by its wide bioactivities, we thus explored to introduce icariin into ADSCs based DMED treatment in the study.

In the present study, the strategy that the icariin administration can efficiently improve the therapeutic potentials of ADSCs for DMED is proposed. To further understand the synergistic effects, *in vitro* oxidative stress test was firstly performed by the introduction of H_2_O_2_ and then the possible underlying mechanism was investigated by related signal protein quantification and inhibiting assays.

## Materials and methods

### Ethical approval and animals

The experiments involving rats were raised and utilized according to the National Institutes of Health Guidelines on the Use of Laboratory Animals. The experimental protocol of rats was approved by the Committee on the Ethics of Animal Care and Use of Chinese PLA general hospital. Sprague-Dawley rats were purchased from Charles River Laboratories (Beijing, China). Animals were separately raised in plastic cages (size: 40 × 30 × 15 cm), maintaining a 12h light-dark cycle (lights on at 07:00 a.m.) with room temperature around 25.0°C, and received food and water ad libitum. Rats were anaesthetized by intraperitoneal injection of pentobarbital sodium (30 mg/kg). Surgeries were all conducted under strictly anesthesia conditions in order to do the best to minimize the possible injury and infection. Carbon dioxide (CO_2_) gas inhalation is used as ethically acceptable method of euthanasia in rat in our study (CO_2_ > 95%).

### Isolation and cultivation of adipose derived stem cells

Isolation and cultivation of adipose derived stem cells were performed according to previous descriptions [29, 30]. Typically, male Sprague-Dawley rats (80-120g) were killed unconsciously; adipose tissues derived from inguen were washed with PBS until the bloodiness was eluted. Then the obtained tissues were minced followed by digestion with collagenase I. After centrifugation, supernatant were discarded and the residual cells were suspended in DMEM with 10% fetal bovine serum, 1% penicillin-streptomycin and 2 mM L-glutamine, and then cultured in humidified atmosphere and 5% CO_2_ at 37°C for 48h. The medium containing free cells were then removed followed by the supplement of fresh culture medium. The culture medium was substituted every 3 days. Cells were passaged when they reached about 90% confluence and passage 3 cells were used in the *in vitro* and *in vivo* experiments. To verify the cellular identity of cells, fluorescence-activated cell sorting was employed via the usage of CD90, CD29, CD34 and CD45 markers.

### Multipotent differentiation

Adipogenic induction: ADSCs at a density of 20,000 cells/cm^2^ were inducted with alpha MEM medium in the presence of 10% FBS, 1% penicillin and streptomycin, 1mM dexamethasone, 500mM 3-isobutyl-1-methylxanthine, 10 mg/ml insulin, and 100mM indomethacin for 21 days. 4% paraformaldehyde was utilized to fix the obtained cells for 30 min at room temperature and then the cells underwent staining with fresh Oil Red O solution for 50 min. The fat droplets were identified under the light microscope.

Osteogenic induction: ADSCs at ad density of 5,000 cells/cm^2^ were inducted with alpha MEM medium containing 100 nM dexamethasone, 10 mM β-glycerophosphate, and 50 mM ascorbic acid-2-phosphate (Wako Chemicals, Richmond, VA) with 10% FBS and 1% penicillin and streptomycin for 21 days. Afterwards, the obtained osteoblasts were treated by 95% ice-cold ethanol for 5 minutes and then stained with 2% Alizarin Red Solution (pH = 4.0). Calcium deposits in cells were observed under light microscopy and orange-red stained areas were recognized as calcium deposits.

### Establishment of diabetic rats

After an overnight fast, 68 Sprague-Dawley rats (10 weeks old) underwent intraperitoneal injection of a citrate acid buffer solution (pH = 4.0) with streptozotocin (STZ; 60 mg/kg; Sigma, St Louis, Missouri) for the purpose of the inducement of diabetes, according to the previously described [31]. After 72 hours, blood samples obtained from tail prick were applicated for blood glucose measurement. The diabetic rats, which tolerated a blood glucose level higher than 200 mg/dL, were selected for the following study.

### Intracavernous administration of ADSCs

Eight weeks after STZ injection, apomorphine (APO, 100 ng/g, Sigma) was used to screen for the ED rats according to Heaton’s method [32]. After the APO subcutaneous injection, 59/68 (86.8%) rats were finally determined as diabetic ED rats. Half of randomly selected ED rats then received left corpus cavernosum (CC) injection of 300 μL PBS solution containing 1×10^6^ ADSCs; All transplantation operations were performed under the same anesthetic conditions. The number of rats was 14 or 15 in each group. Prior to aseptic implantation, all rats were anesthetized with ketamine (30 mg/kg) and xylazine (4 mg/kg). Half of the ADSCs treated or non-treated ED rats were further given with PBS with/without icariin (5 mg/kg) through gastric perfusion, respectively. Icariin (purity >98%) was purchased from Beijing HWRK Chem Co., LTD, PR China.

### *In vivo* viability of ADSCs

DiI (Invitrogen) fluorescence staining (red), a common label reagent used for detecting implanted alive cells *in vivo*, was used to label ADSCs, according to the manufacturer’s protocol. Post-transplantation for 1 week, the frozen section of corpus cavernosum was harvested and underwent observation in fluorescent microscope.

### Erectile function assessment

The value of mean arterial pressure (MAP) and intracavernosal pressure (ICP) at the 4th week were utilized for the assessment of erectile function according previous reports [33]. In brief, after anesthetization, the exposed left carotid artery of rats was then cannulated by a PE-50 tube filled with 250 IU/ml heparinized saline, connecting to a pressure channel in order to constantly monitor MAP. As for the detection of ICP, both the penile crus and cavernous nerve (CN) of rats were exposed by a lower midline abdominal incision. A heparinized (heparin 250 IU/ml) 25-G needle linked to another pressure transducer for the purpose of recording physiological changes was subsequently inserted into left crura of rats. The electrostimulation adjacent to CN was conducted utilizing a bipolar electrode and controlled by an electrical stimulator (ShangHai Biowill Co., Ltd. Shanghai, China), which was capable of generating monophasic rectangular pulses (a fixed width of 0.2 ms, 1.5 mA, frequency at 20 Hz, and duration of 50 s). Every ten minutes, the stimulation was performed for three times and continuous simultaneous recordings of MAP and ICP employed a PC Lab (ShangHai Biowill Co., Ltd. Shanghai, China) signal process system. The highest value of ICP was selected for statistical analysis. The calculated ratio of maximum ICP/MAP was used to display the erectile responses.

### Histology and immunohistological analysis

The central parts of the rat penile shafts (~2.5 mm) were cut and fixed in 4% paraformaldehyde. The harvested tissue were then dehydrated by an ethanol gradient and embedded in paraffin. 5 μm paraffin-embedded sections were prepared for Masson’s trichrome staining to assess the collagen deposition (tissue scarring). For the next immune staining with vWF (Sigma) and αSMA (Sigma) antibodies, images of 100× magnification were utilized (Santa Cruz Biotechnology, Inc.). Quantitative image analysis employed the method of computerized densitometry via the ImagePro program (version 6.3) coupled to a Leica microscope. Then, the expression levels of markers in each were standardized as the ration to control. Ten sections were counted for every penile tissue of rats and six fields were calculated from each section.

### Incubation of ADSCs upon *in vitro* oxidative stress

The *in vitro* oxidative stress conditions were fabricated through the addition of H_2_O_2_ into culture medium. Then, the as-prepared AMCSs were cultivated in the medium containing discrete H_2_O_2_ concentrations of 0, 25, 50, 100, and 200 μM, respectively. After 12 h incubation, the treated cells were collected and subsequently stained with PI for the following cell viability assays via flow cytometry.

The anti-oxidative protective effects of icariin on ADSCs were confirms through the introduction of different concentrations of icariin (0, 25, 50, 100, 200 μM) into the culture medium. After 12 h cultivation, the cell death was assessed by similar methods.

### Determination of intracellular ROS levels

Intracellular ROS levels were analyzed by dihydroethidium (DHE) staining methods. Briefly, 1 mM DHE was mixed with ADSCs. After incubation at 37°C for 15–25 min, the mixtures were gently washed with PBS buffers for 3 times. The cells were then collected, and underwent the measurement of their emission through a FACS can flow cytometer (BD Biosciences).

### Measurements of SOD activity and the leakage of LDH

The SOD and LDH assays were performed as the manufacturer’s instructions. Typically, for the determination of SOD activity, the treated ADSCs were washed with 4°C PBS for 3 times and resolved in 100 mM PBS containing 1% Triton X-100 for complete lysis. After centrifugation at 12000 rpm at 4°C for 20 min, the supernatant was collected for the activity assay according to the manufacturer’s protocols. For the LDH leakage assay, the level of LDH was determined using LDH Kit (Jiancheng, China). The supernatants of the treated ADSCs were harvested for the LDH measurement and LDH activity was then calculated by the absorbance at 440 nm utilizing a UV–visible spectrophotometer (Beckman DU-640B, USA).

### Western blotting

The lysis of ADSCs exploited Laemmli Sample Buffer (Bio-Rad). After centrifugation at 4°C, proteins components were attained and protein levels were determined by BCA^TM^ Protein Assay Kit (Thermo Scientific). Afterwards, proteins were loaded for sodium dodecyl sulfate polyacrylamide gel electrophoresis (SDS-PAGE). After the obtained discrete proteins were then transferred to nitrocellulose membranes, primary antibodies against p-Akt(abcam), p-STAT3(abcam), cleaved Csp3 (abcam), Bax (abcam), or Bcl-2 (abcam) were incubated over night at 4°C. Finally, the corresponding secondary antibodies were incubated for another 1 h at room temperature. GAPDH was used as the internal reference.

### Statistical analysis

Statistical values in this study were expressed as mean±standard deviation (SD). *P* values<0.05 were taken as statistically significance. Comparisons of ICP, ICP/MAP, and immunofluorescence staining analysis among the groups were conducted by using 1-way ANOVAs followed by Tukey's post hoc test for multiple pair-wise examinations.

## Results

### *In vivo* survival of ADSCs and diabetic erectile function assessment

**Fig 1:**
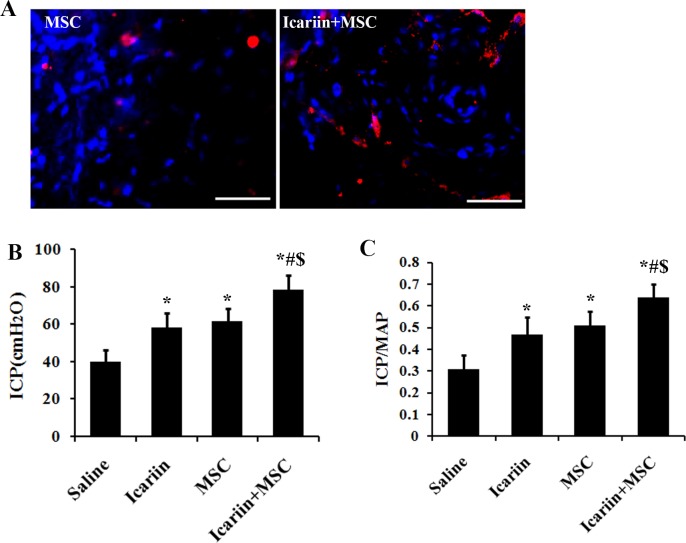
Icariin promotes the *in vivo* survival of ADSCs and the ADSCs induced recovery of erectile function of DMED rats. A) The *in vivo* survival of ADSCs before and after administrating icariin was assessed by Dil staining. B) and C) ICP and MAP measurements were employed in the assessment of erectile function of DMED rats. The rats were injected with saline or ADSCs followed by feeding up with saline or icariin for 4 weeks. The ICP value and the ratio of total ICP to MAP were calculated, n = 7. * p<0.01 compared with the control saline treated group, # p<0.01 compared with icariin treated group. $ p<0.01 compared with the ADSCs treated group. Scale bar: 50 μm.

To track the survival of transplanted cells, ADSCs were labeled with DiI pre-transplantation and one week post transplantation, the survival of transplanted ADSCs were detected by observation with fluorescent microscope. As showed Fig 1A, compared with ADSCs group, significantly more DiI-positive cells (red) were observed in ADSCs+icariin group, indicating that ADSCs survival was significantly improved due to the application of icariin. To evaluate functional benefits of intracavernous transplantation of ADSCs combined with the gastric administration of icariin for the diabetic ED, we employed representative ICP measurements through electrostimulation of the cavernous nerve (CN) (Fig 1B and 1C). Rats that received merely ADSCs or icariin treatment exhibited a significant trend of improved ICP and maximum ICP/MAP ratio in response to CN stimulation compared with saline treated groups (*p*<0.01). Moreover, rats that received ADSCs transplantation and 4 days gastric gavage of icariin simultaneously were observed with significantly better measuring data than those received alternative treatment alone (*p*<0.01), suggesting the *in vivo* practical feasibility of the combined administration of ADSCs and icariin.

### Damage of blood vessels in cavernous tissue

**Fig 2:**
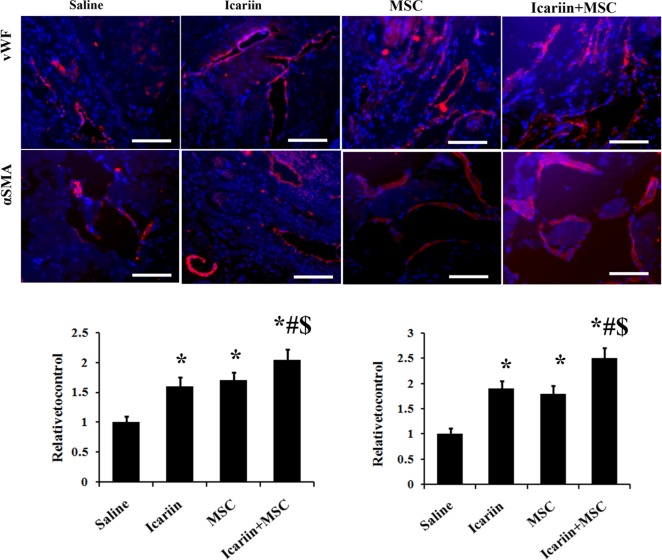
The treatments of icariin and ADSCs enhance the expression of vascular markers in DMED rats. The rats were injected with saline or ADSCs followed by feeding up with saline or icariin for 4 weeks. Top panel: representative images of vWF and α-SMA-positive ADSCs (red) in corpus cavernosum of DMED rats receiving saline, ADSCs and icariin treatment. Bottom panel: Ratio of vWF and α-SMA identified in corpus cavernosum expressed as relative expression to the control group. Immunofluorescent staining analysis of cavernous tissue used vWF and α-SMA antibodies in sections of diabetic rats corpus cavernosum. DAPI staining and vessel: dash line; n = 7, scale bar: 50um, * p<0.01 compared with the control saline treated group, # p<0.01 compared with icariin treated group. $ p<0.01 compared with the ADSCs treated group.

To understand whether ADSCs transplantation along with 4 days feeding on icariin might have an impact on angiogenesis in the STZ induced diabetic rats, we utilized immunofluorescence staining with vascular markers for the investigation of vascular restoration. As showed in Fig 2, significantly fewer cells expressed endothelial markers (vWF) in the saline-treated rats than that in the ADSCs or icariin treated rats (*p*<0.01). However, the combination of the ADSCs and icariin treatment significantly promoted the endothelial contents compared with the separated treatment groups (*p*<0.01).

When it comes to the vascular smooth muscle, α-SMA expression was measured. The cavernous expression of α-SMA was significantly higher in the ADSCs or icariin treated rats than that in the saline treated ones (Fig 2) (*p*<0.01), while the α-SMA in ADSCs and icariin jointly administrated groups were significantly higher than that in ADSCs injected or icariin perfused rats (*p*<0.01). The results mentioned above suggested a significantly augmented vascular protection or angiogenesis of the combined treatment of ADSCs and icariin.

### Collagen deposition

**Fig 3:**
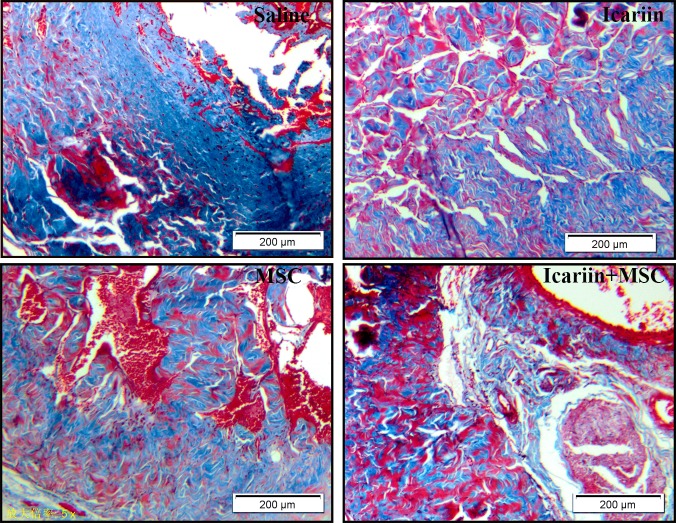
Results of histological analysis of collagen deposition. Masson’s trichrome staining to assess the collagen deposition which indicated tissue scarring. Smooth muscles were stained in red while collagen fibers were in blue, n = 7. Scale bar: 200 μm.

Masson’s trichrome staining was utilized to assess the collagen deposition, indicating tissue scarring. As shown in Fig 3, abundant collagen deposition in saline group was observed. However, in animals receiving ADSCs injection or icariin treatment, red staining parts expended largely and contrarily blue staining areas shrank, demonstrating the restoration of smooth muscles. When combined, the two methods considerably enhanced the recovery of the smooth muscles in corpus cavernosum, compared with the ADSCs or icariin solely treated groups.

### Characteristics of ADSCs

**Fig 4:**
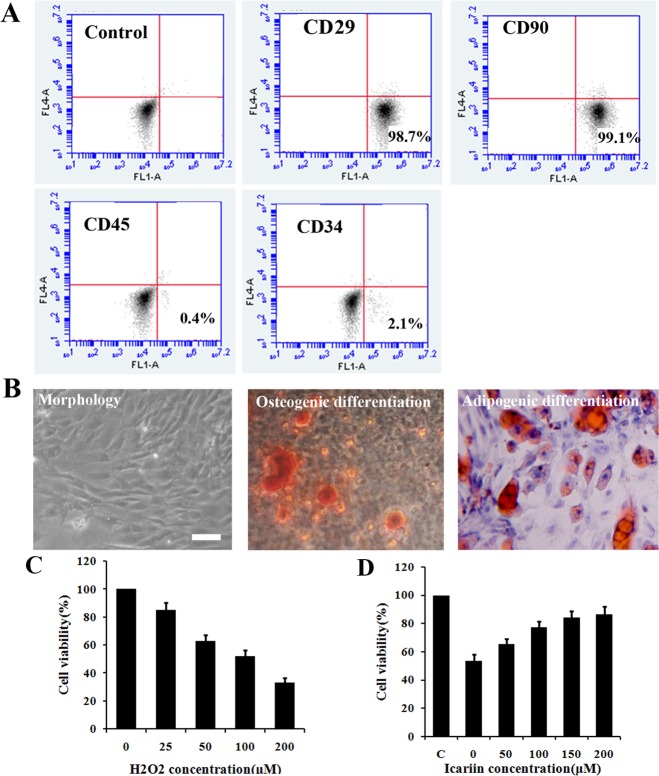
Characteristics of ADSCs and effects of H_2_O_2_ and icariin on the viability of ADSCs and H_2_O_2_ treated ADSCs, respectively. A) Flowcytometry demonstrated that most ADSCs expressed CD90, CD29 but less CD45, CD34. B) Morphology of ADSCs and multipotency of ADSCs, differentiating into adipocytes stained by Oil red O and osteocytes stained by Alizarin red S (Scale bar: 100 μm). C) Cells were treated with 0, 25, 50, 100, and 200 μM H_2_O_2_ for 12 h followed by PI staining with flow cytometry to determine cell viability, n = 7. D) Cells were treated with 0, 50, 100, 150 and 200 μM icariin under 100 μM H_2_O_2_ containing medium for 12 h incubation. Cell viability was determined using PI staining, n = 7.

To better verify our results, we next systematically characterized the immune phenotype and differentiation potential of ADSCs. As showed in Fig 4A, the majority of the isolated cells expressed CD29 and CD90, while negative for CD31 and CD45. The representative morphology image of ADSCs was presented in Fig 4B. The multipotency of ADSCs was examined by differentiation induction into adipocytes, containing oil droplets stained with Oil Red O, and osteoblasts, producing calcium stained with Alizarin Red S. (Fig 4B)

### Effects of icariin on the ADSCs viability under *in vitro* oxidative stress condition

The introduction of H_2_O_2_ into medium was used to establish *in vitro* artificial oxidative stress culture environment. As illustrated in Fig 4C, the viability of ADSCs significantly decreased along with the increasing of H_2_O_2_ concentration. When the concentration of added H_2_O_2_ reached 100 μM, only about 50% cells can survive, which was also selected as the model oxidative stress condition to evaluate the protective effects of the following anti-oxidant. Moreover, the 200 μM H_2_O_2_ containing cultural condition resulted in 60–70% cells death. These results clearly demonstrated that the oxidative stress had a potent impact on the fate of ADSCs and thereby agents with anti-oxidative properties can possibly improve the suffering. Icariin has long been evidenced to be a powerful anti-oxidant against oxidation induced cellular damages. As predicted, the 100 μM H_2_O_2_ induced decrease of ADSCs viability can be considerably attenuated by the addition of icariin. Moreover, as shown in Fig 4D, the growing levels of icariin can increasingly enhance the ADSCs survival under the oxidative stress condition. When the concentration of icariin arrived at 150–200 μM, the recovery of the viability of cells stabilized at 80% more or less.

### Study of oxidative stress injury

**Fig 5:**
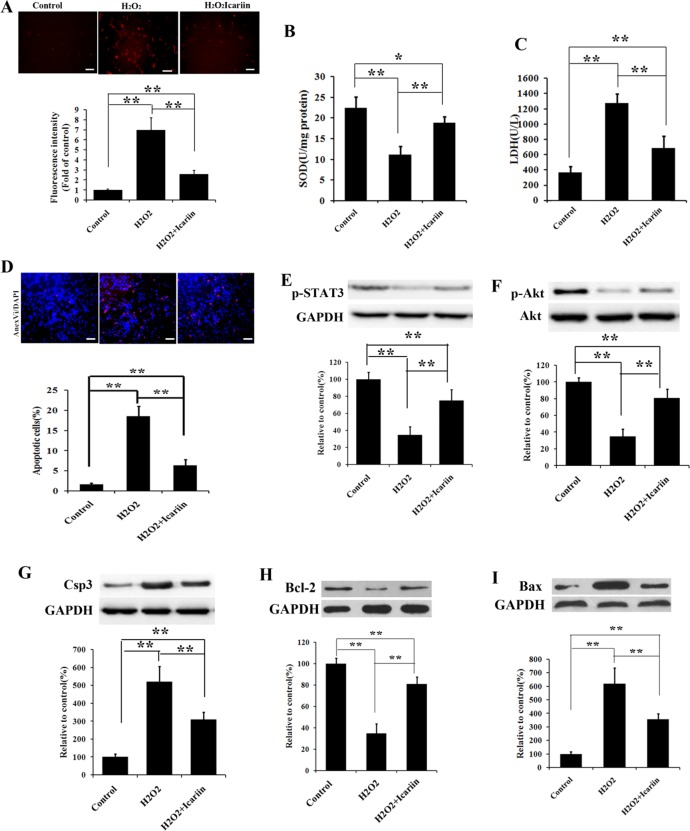
Icariin prevents ADSCs from oxidative stress-induced cell damage and apoptosis. (A) Cells were pretreated with 100 μM H_2_O_2_ and icariin for 12 hours, and then targeted with superoxide indicator DHE. The level of ROS was quantified by the amount of cellular ethidium formation. The produced fluorescence intensity was analyzed through a fluorescent microplate reader, n = 7. (B, C) After administration without or with H_2_O_2_ and/or icariin for 12 h, the activity of SOD and the leakage of LDH were evaluated according manufacturer’s protocols, n = 7. (D) Cells were treated under the same conditions as in (B, C) and apoptosis was evaluated by Annexin V staining, n = 7. (E-I) Cells were administrated as described in (B, C) and relative changes of p-STAT3, p-Akt, Csp3, Bcl-2, and Bax levels were measured by Western blotting. GAPDH was utilized as the loading reference. All data are expressed as mean ± SD, *p < 0.05, **p <0.01. Scale bar in A, D equals 50 μm.

To further understanding the icariin promoted recovery of oxidative stress cellular damage, the staining methods of DHE, which selectively reacted with ROS and subsequently produced red emission, was employed as the indicator for the ROS measurement. The high level of cellular ROS was reported to contribute to the apoptotic cell death [34]. As shown in Fig 5, 100 μM H_2_O_2_ treated group emitted stronger red light compared with the control group. After introduction of icariin, the red emission of DHE triggered by ROS significantly attenuated. The quantitative results of the red emissive intensity clearly reflected the effects of icariin upon the ROS levels. The addition of icariin significantly reduced about 64% of the ROS level produced by the model oxidative stress, suggesting the promising potential of icariin for relieving the intracellular oxidative stress.

To confirm our assumption, we then examined another two important oxidative stress related indexes, SOD activity and the leakage of LDH. As shown in Fig 5, the administration of H_2_O_2_ caused more than half loss of SOD activity and the levels of the leaking LDH in the oxidative stress group were over 3 fold higher than the control. This situation can be effectively offset by the addition of icariin. Upon the introduction of icariin, the SOD activity can reach about 85% of the original state, while the leakage of LDH were also fall to about 1.5 fold levels of the control group. In the concern of the above results, we can demonstrate that H_2_O_2_ can trigger the oxidative stress damage of ADSCs and icariin possesses the protective effects of the injured cells against oxidative stress.

### Apoptosis assays

A high oxidative stress can cause destructive and lethal cellular effects, such as apoptosis. To evaluate the possible cellular effects induced by H_2_O_2_, Annexin V staining was utilized to assess the apoptosis of ADSCs. Fig 5 expressly revealed that the percentage of apoptotic cells displayed significantly almost 9 fold increase after the introduction of H_2_O_2_ and the following addition of icariin can promote the survival of ADSCs with merely about 3 fold increase compared with the control group, suggesting icariin can weaken the apoptotic effects induced by high oxidative stress.

To attain a deeper insight into the mechanism of protective effects of icariin on oxidative stress induced ADSCs apoptosis, we examined the protein expression of relative signal pathways via western blotting methods. As showed in Fig 5, compared with the control group, the group undergoing H_2_O_2_ treatment exhibited a significant decrease of the expression of p-STAT3. Correspondingly, the level of p-Akt was also descended. However, upon the introduction of icariin, the expression of p-STAT3 and p-Akt received considerable enhancement, indicating a positive relationship between the addition of icariin and the expression of the two proteins. Subsequently, we measured the expression of apoptotic indicators, including Csp3, BcL-2, and Bax. As shown in Fig 5, similarly, the expression of apoptosis positive related protein, Csp3 and Bax, in the H_2_O_2_ treated group was higher than that in the control group, and contrarily the expression of apoptosis negative related protein, BcL-2, was accordingly suppressed. Upon the administration of icariin, the up-regulation of Csp3 and Bax of H_2_O_2_ treated cell were significantly attenuated, while the expression of anti-apoptotic protein BcL-2 was significantly promoted.

### STAT3 inhibition offsets the protection of icariin on ADSCs from oxidative stress

To confirm whether the activation of p-STAT3 possesses antioxidatively protective effects on ADSCs, we additionally introduced p-STAT3 inhibitor, LY5, into the icariin treated ADSCs for the purpose of suppressing the activity of p-STAT3. As shown in S1 Fig, the level of p-STAT3 expression in H_2_O_2_ treated cell significantly increased after the introduction of icariin. And the following addition of LY5 led to the descending of the level of p-STAT3, which was similar to the expression of the merely H_2_O_2_ treated cells. Accordingly, the icariin promoted regulations of apoptosis related signal proteins were also offset by the introduction of p-STAT3 inhibitor. As the result of the inhibition of p-STAT3, the expression of Csp3 and Bax was significantly enhanced, while the expression of anti-apoptosis protein, BcL-2, was in contrast down-regulated to the level of that in the merely H_2_O_2_ treated ADSCs. These aforementioned results indicated that ADSCs protection of icariin against oxidative stress was through activation of STAT3.

### STAT3 activation-mediated ADSCs protection of icariin was regulated by PI3K/Akt signaling pathway

To further understanding the association between the up-stream signal PI3K/Akt and p-STAT3 as well as the “down-stream” cell protection, we thus designed the PI3K/Akt signaling pathway inhibition experiment. The PI3K inhibitor, LY294002, was given after the administration of icariin on the H_2_O_2_ treated ADCSs. As illustrated in S2 Fig, icariin significantly up-regulated the expression of p-Akt, which was subsequently suppressed by the addition of LY294002. The result of p-STAT3 expression showed that the activation of p-STAT3 was inhibited along with the inhibition of the icariin driven up-regulation of p-Akt, suggesting a positive relationship between p-STAT3 and p-Akt. In addition, the measurement of apoptosis related protein displayed similar results to that of the p-STAT3 inhibition assay. And, that is, the icariin induced regulation of Csp3, Bax and BcL-2 expression were reversed to the level of that in the control group.

## Discussion

Nowadays, increasing men have suffer the trouble from ED which has a severe impact on human health and life [35]. In recent years, among the fights against ED, stem cell transplantation therapy occurred and has been regarded as a promising weapon against ED. Mesenchymal stem cells derived from human adipose tissue (ADSCs) received particular concerns as the result of their featured biological benefits, including abundance of autologous sources, ease of isolation and expanding [36]. Meanwhile, several studies demonstrated that the implantation of ADSCs can significantly recovered the diabetes-induced erectile function [17, 18], supporting the feasibility of stem cells therapy for the regain of erectile function. Subsequently, many efforts in the enhancement of the therapeutic effects of ADSCs have been proposed. And our previous study demonstrated hypoxia treated ADSCs achieved a better restoring effect on DMED, indicating that suitable training for ADSCs was pleasurable direction to acquire therapeutic enhancement. Besides, co-treatment methods, currently, received more attentions, due to their feasible and established operation procedure, relatively sound mechanism and high possibility to attain ideal enhanced effects. Thus, based on ADSCs treatment, we next conducted the study on a combined strategy for DMED therapy.

Herein, icariin was introduced in the ADSCs based co-treatment strategy for the first time. Icariin is the primary active component of the Traditional Chinese herb, *Epimedium*, and has an appreciable protective effect on cells in a wide range, including anti-inflammatory, antioxidant, anti-depressant and neuroprotective activities. More importantly, icariin has showed the capability of restoring DMED, albeit ambiguous mechanism [37].

To assess the therapeutic effects of icariin treated ADSCs to the DMED, MAP and ICP measurements were utilized for the evaluation of erectile function preliminarily. The testing data clearly demonstrated that the introduction of icariin can significantly enhanced the erectile function restoration induced by ADSCs transplantation, suggesting the potential practical application for the treatment of diabetic ED. Apart from the functional recovery, ADSCs have been evidenced to possess the capability for the vascular and nerve structural regeneration in the corpus cavernosum of the ED sufferer. Upon 4 weeks administration of icariin after transplantation, the in situ tissue structure was also analyzed by Masson’s trichrome staining and immunology staining the sections with vascular endothelial marker (vWF) and vascular smooth muscle marker (α-SMA). Those staining results conformed to the former data, further demonstrating that the combined treatment of ADSCs transplantation and icariin gastric gavage can considerably enhance the improvement of diabetic ED in the aspect of both function and structure. The benefit of icariin on stem cell-based therapy of ED suggested that combination of suitable drugs and stem cells may be more effective for ED. At present, PDE5 inhibitor was clinically a standard therapy for ED and it worked well too for diabetic ED model as we used in the present study [38], but the efficacy of the drug was not always satisfied for all ED patients [9]. It could be supposed that combination PDE5 inhibitor and stem cells may work better than PDE5 inhibitor alone, which deserved further investigation in future.

As is known, one of the most featured pathological events in diabetic ED is oxidative stress, which not only severely impaired the blood vessels and adjoining tissues, but also led to mass mortality of exogenous transplanted stem cells, thus limiting the therapeutic effects of transplanted stem cells [39, 40]. Icariin has been reported to serve as excellent antioxidant [41]. Therefore, we supposed that the reason why icariin could enhance the therapeutic efficacy of ADSCs on ED may be that the introduction of icariin significantly improved the tissue local microenvironment, such as reducing ROS and oxidative stress in cavernoursal region, thus lowering the death of transplanted MSC. In our *in vivo* study, we observed that the introduction of icariin produced a much more amount of living ADSCs, potently supported our hypothesis. Based on aforementioned hypothesis, we further designed and conducted a series of *in vitro* experiments by employing H_2_O_2_ to simulate the oxidative microenvironment, aiming to determine whether anti-oxidative protection of icariin wan one of the potential mechanisms. After introducing H_2_O_2_, we observed that H_2_O_2_ induced oxidative stress could significantly increase the death of ADSCs, suggesting that the ADSCs engrafting into DMED corpus cavernosum were more difficult to live through the relative high oxidative stress, limiting the therapeutic capacity of ADSCs. Additionally, several previous studies have demonstrated that icariin was useful in prevention of oxidative stress-induced cells damage and death. Therefore, this finding hinted us that the synergistic effects induced by icariin were likely associated with the improvement the viability of cells [42–44]. Then, we introduced icariin into the ADSCs *in vitro* oxidative stress cultural system. Indeed, the introduction of icariin considerably promoted the viability of the cells, indicating icariin as a promising agent for the therapeutic enhancement of ADSCs transplantation. The following oxidative stress induced cellular damage assessment further confirmed that icariin was able to increase the amount of living ADSCs as the result of the recovery of oxidation related injury.

To deeper understand the underlying mechanism, we performed western blotting assay on a series of proteins that were related to the oxidation induced apoptotic death. We found that the abnormal secretions of the apoptotic indicators owing to oxidative stress were significantly restored after the addition of icariin and the restored expression involved un-regulation of p-STAT3 [45], p-Akt [46] and BcL-2 [47], and down-regulation of Csp3 [47] and Bax [47]. In the view of the obtained staining and blotting data, we thereby speculated that cellular oxidative stress induced apoptosis was mainly through the inhibition of PI3K/Akt-STAT3 signaling pathway. Afterwards, the experiments on inhibiting signal proteins were conducted and the results were exactly consistent with our assumption. The icariin driven restoration of the expression of STAT3 and its down-stream proteins can be abrogated by the introduction of STAT3 inhibitor. The down-regulation of STAT3 facilitates to the procedure of apoptosis, resulting in the increase of the level of apoptotic proteins and the descending of the level of anti-apoptotic proteins. Furthermore, the offset expression level of STAT3 can be suppressed by inhibiting its up-stream regulators, PI3K/Akt signal species. By introducing the LY294002 which is specified for the inhibition of PI3K/Akt, the expression of STAT3 was significantly down-regulated and the expression of other assessed proteins was similar to the result of STAT3 inhibition assay.

However, albeit plausible results with DMED improvement via the administration of ADSCs and icariin, the icariin given by gastric gavage could be transformed to its metabolic derivatives such as icariside II in rats *in vivo* and thus weather icariside II and other derivatives also emerged in ADSCs corpus cavernosum was still unclear. In addition, the animal model used in the present study was Sprague Dawley rat, an outbred strain. As is known, minor histocompatibility differences may exist in such animals. An inbred strain, such as Wistar rat, may further minimize the potential immunologic impact on results. However, low immunogenicity was a typical property of MSCs, which has been demonstrated by several studies previously [48–50]. This was also one of the important reasons that MSCs were considered the promising seeding cells in regenerative medicine. In the field of MSC-based therapy, both SD and Wistar rats were usually used as animal models, but no study has observed immunologic difference between the two models after receiving homologous transplantation, nor did we in the present study. Furthermore, rats were also frequently used as models to receive human MSC transplantation just because of the low immunogenicity of MSCs [51, 52]. Therefore, it was supposed that minor histocompatibility differences among the SD rat individuals may not produce significant impact on the results of the present studies.

In summary, we investigated the combined treatment of ADSCs and icariin for DMED. The *in vivo* results indicated that the intake of icariin contributed to a significant improvement in the therapeutic effects of ADSCs for DMED. To understanding the possible mechanism, we first built an *in vitro* oxidative stress model to imitate DMED corpus cavernosum microenvironment and we found that the introduction of icariin can promote the viability of ADSCs. Then, the underlying mechanism was further explored by staining and blotting methods. Through the comprehensive examination of the expression of apoptosis related proteins, the regulation of PI3K/Akt-STAT3 signaling pathway by icariin was responsible for the restoration of the oxidative stress induced apoptotic death. All in all, this research provides a novel therapeutic strategy for promoting the treatment of DMED with ADSCs, suggesting a promising potential in the future clinical practice.

## Supporting information

S1 Fig Blockade of STAT3 by LY5 attenuates icariin induced restoration of oxidative stress-mediated cell apoptosis. ADSCs were treated with or without H2O2, icariin and LY5, and then the expression of p-STAT3, Csp3, Bcl-2, and Bax levels were detected by Western blotting. GAPDH was utilized as the loading reference. All data are expressed as mean ± SD, * p<0.01 compared with H2O2 treated group, # p<0.01 compared with icariin treated group.
(DOCX)

S2 Fig Blockade of PI3K/Akt by LY294002 attenuates icariin induced restoration of oxidative stress-mediated cell apoptosis. ADSCs were treated with or without H_2_O_2_, icariin and LY294002, and then the expression of p-Akt, p-STAT3, Csp3, Bcl-2, and Bax levels were detected by Western blotting. GAPDH was utilized as the loading reference. All data are expressed as mean ± SD, * p<0.01 compared with H_2_O_2_ treated group, # p<0.01 compared with icariin treated group.
(DOCX)
