# Supplementary material for: Combination of mesenchymal stem cell injection with icariin for the treatment of diabetes-associated erectile dysfunction
Source: PLoS One. 2017 Mar 28;12(3):e0174145. doi: 10.1371/journal.pone.0174145 (PMC5369760; doi:10.1371/journal.pone.0174145)
Supplement: S2 Fig — ADSCs were treated with or without H2O2, icariin and LY294002, and then the expression of p-Akt, p-STAT3, Csp3, Bcl-2, and Bax levels were detected by Western blotting. GAPDH was utilized as the loading reference. All data are expressed as mean ± SD, * p<0.01 compared with H2O2 treated group, # p<0.01 compared with icariin treated group. (DOCX) [file pone.0174145.s002.docx]

**Figure S2**


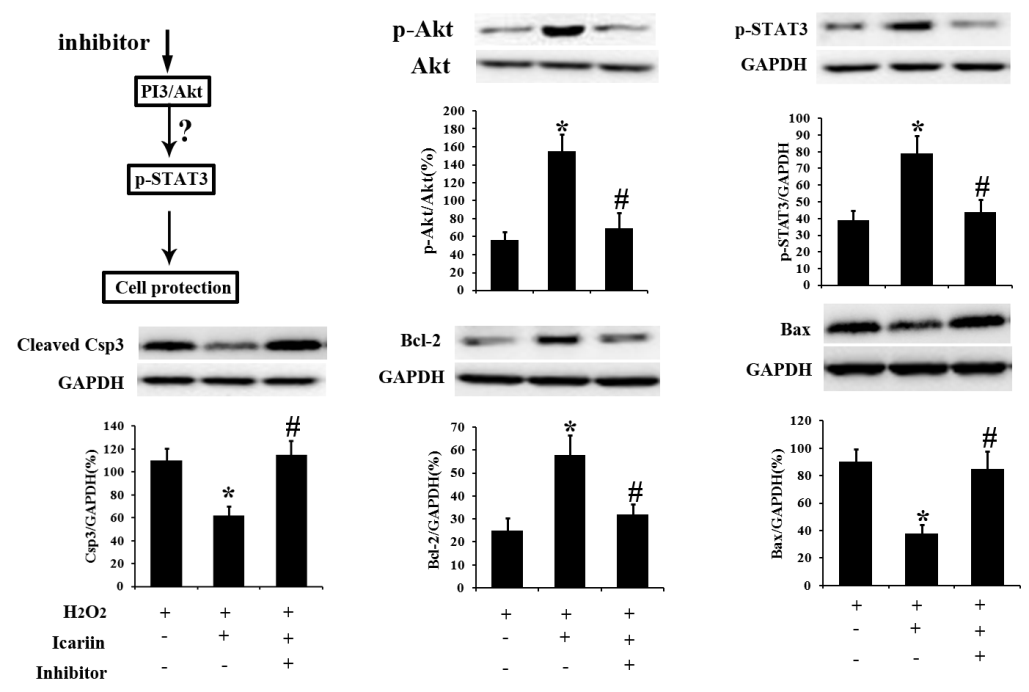


**Fig S2. Blockade of PI3K/Akt by LY294002 attenuates icariin induced restoration of oxidative stress-mediated cell apoptosis.** ADSCs were treated with or without H_2_O_2_, icariin and LY294002, and then the expression of p-Akt, p-STAT3, Csp3, Bcl-2, and Bax levels were detected by Western blotting. GAPDH was utilized as the loading reference. All data are expressed as mean ± SD, * p<0.01 compared with H_2_O_2_ treated group, # p<0.01 compared with icariin treated group.
